# Cancer Therapeutics Following Newton’s Third Law

**DOI:** 10.4172/2168-9652.1000e145

**Published:** 2016

**Authors:** Ali S. Arbab, Meenu Jain, Bhagelu R Achyut

**Affiliations:** Tumor Angiogenesis Lab, Cancer Center, Augusta University, Augusta 30907, GA, USA

## Abstract

Cancer is a wound that never heals. This is suggested by the data produced after several years of cancer research and therapeutic interventions done worldwide. There is a strong similarity between Newton’s third law and therapeutic behavior of tumor. According to Newton’s third law “for every action, there is an equal and opposite reaction”. In cancer therapeutics, tumor exerts strong pro-tumor response against applied treatment and imposes therapeutic resistance, one of the major problems seen in preclinical and clinical studies. There is an urgent need to understand the tumor biology of therapy resistant tumors following the therapy. Here, we have discussed the problem and provided possible path for future studies to treat cancer.

## Editorial

There is no doubt that cancer is a smart entity, which is evident by several treatment failures in preclinical and clinical trials. In part, host genetic mutations are known to be responsible for the limited success [1]. However, in most of the cases, following therapy, tumors itself acquires resistant properties. In this case, tumor is initially sensitive to the applied treatment and evolves itself to counteract the anti-tumor effects of drug. On the other hand, de novo resistance is the part of primary refractoriness to a therapy that should have been effective based on the underlying biology or genetics. According to Newton’s third law “for every action, there is an equal and opposite reaction”. In cancer therapeutics, tumor exerts strong pro-tumor response against applied treatment. Most of the therapies with short and transient benefit (measured in weeks or months), have witnessed relapse of malignant tumor growth [2,3]. Resistant tumors are characterized by hyper-vascularity and hyper-invasiveness, for example; breast cancer and glioblastoma [4,5]. Tumor cells become quiescent by reprograming into mesenchymal phenotypes. In epithelial tumors, this phenomenon is well evident and known as epithelial to mesenchymal transition (EMT) [6,7]. In addition, resistant tumor secretes several immunomodulatory signals in the form of secreted factors such as chemokines and growth factors [8]. By doing so, tumor modulates immune cells and governs to impose pro-tumor properties. Heterogeneous population of tumor associated macrophages (TAMs) and regulatory cells are the most abundant pro-tumor and immune-suppressive immune cells known, which contribute to tumor recurrence following therapy [9]. Recently, much attention has been given to the bone marrow derived cells (BMDCs), which is the host component. Ample amount of data suggests that immune cells are derived from bone marrow compartment and resistant tumor recruits these immune cells on regular basis either at the time of tumor initiation, progression or metastasis at the distant organs [5]. Studies, including reports from our lab support that recruitment of bone marrow-derived myeloid cells, especially; myeloid derived suppressor cells (MDSCs) are critical in therapeutic resistance [10-14]. We believe that MDSCs acquire vasculogenic properties to provide vasculature support to the transiently shrink tumor. Our previous study illustrates that depleting bone marrow-derived myeloid cells through CSF1R blockade, significantly decreased recruitment of BMDCs from bone marrow to the tumor site. In addition, CSF1R blockade decreased tumor associated MDSCs and reduced tumor growth. Thus, targeting TAMs is crucial in avoiding therapeutic resistance [13,14]. At this point, we need more in-depth understanding of resistant tumors and their microenvironments through detailed mechanistic studies. We have to evolve our approaches to monitor such resistance at the earlier period of therapy and during the therapy.

In summary, most of the cancer therapies are limited by the development of drug resistance and to bypass this circumventing resistance is our priority in the era of personalized medicine. [15]. Our understanding of the cellular and molecular mechanism(s) of drug resistance has been increasing day-by-day. Discovering biomarkers of therapeutic resistance could be a good tool to find resistant patients, during the therapy. In addition, using new experimental approaches coupled with the systematic genomic and proteomic technologies would identify novel targets [12].
